# Challenges in current nursing home care in rural Germany and how they can be reduced by telehealth - an exploratory qualitative pre-post study

**DOI:** 10.1186/s12913-021-06950-y

**Published:** 2021-09-06

**Authors:** Susann May, Kai Jonas, Georgia V. Fehler, Thomas Zahn, Martin Heinze, Felix Muehlensiepen

**Affiliations:** 1 Center for Health Services Research, Brandenburg Medical School Theodor Fontane, Seebad 82/83, 15562 Rüdersdorf, Germany; 2 bbw Hochschule Berlin, Berlin, Germany; 3 Department of Psychiatry and Psychotherapy, Immanuel Klinik Rüdersdorf, Rüdersdorf, Germany; 4 Faculty for Health Sciences Brandenburg, Potsdam, Germany

**Keywords:** Telehealth, Video consultation, Rural areas, Physician shortage, Nursing homes

## Abstract

**Background:**

Telemedical care of nursing home residents in Germany, especially in rural areas, is limited to a few pilot projects and is rarely implemented as part of standard care. The possible merits of implementing video consultations in longer-term nursing care currently lack supporting evidence. In particular, there is little documentation of experiences and knowledge about the effects and potential benefits of the implementation in presently existing structures. The goal was to assess the effect of implementing medical video consultations into nursing home care addressing the following research questions:

- How is medical care currently provided to nursing home residents, and where do problems in its implementation arise?
- How can video consultations be used to reduce difficulties arising in everyday care?
- How does implementation of video consultations impact day-to-day nursing home care delivery?

**Methods:**

Twenty-one guided interviews (pre-implementation *n* = 13; post-implementation *n* = 8) were conducted with a total of 13 participants (physicians, nurses and medical technical assistants). Narratives were analysed using qualitative content analysis. The results were contrasted in a pre-post analysis.

**Results:**

Challenges described by the participants before implementation included a requirement for additional organisational and administrative efforts, interruptions in the daily care routine or delayed treatments, and risk for loss of patient-relevant information due to process diversity. After implementation, communication was facilitated upon introduction of assigned time slots for video consultations. Clinical information was less likely to be lost, additional work was spared, and medication and therapeutic and assistive devices were provided more quickly.

**Conclusions:**

Telehealth cannot replace physical, in-person visits, but does offer an alternative form of service delivery when properly integrated into existing structures. Our results suggest that the use of video consultations in nursing homes can reduce the burden and additional workload, and increase the efficiency of care provision for nursing home residents. Video consultations can complement in-person visits to nursing homes, especially to address the shortage of medical specialists in rural areas in Germany. To promote implementation and acceptance of video consultation in nursing homes, we need to increase awareness of its benefits and undertake further evaluation of video consultations in nursing home care.

**Supplementary Information:**

The online version contains supplementary material available at 10.1186/s12913-021-06950-y.

## Background

Telehealth interventions, particularly via video consultations, are emerging as a relevant contribution to medical care. Telehealth has the potential to expedite access to care and to facilitate “healing at a distance” of individuals separated geographically from care providers [1]. In rural areas, various barriers impede access of patients to their physicians. On the one hand, traveling to a physician can impose physical, psychological, and financial hardships on seriously ill rural patients, especially among the elderly [2]. On the other hand, barriers due to access and high volume of patients in the practice can preclude timely in-person home visits by physicians in a rural setting. In rural and remote communities, the impeded access due to health services can contribute to poorer health outcomes [3]. In this setting, telehealth has become an important tool for improving health care [4]. Implementing video consultations into standard practice could further address the difficulties arising from physician shortages in rural areas of Germany [5, 6].

Study results indicate that implementation of telehealth interventions is an effective way to deliver health services to rural communities, with positive influences on the quality, coordination, and organisation of health care services [7], bringing a reduction in unnecessary emergency admissions and hospitalizations rates [8–10]. Furthermore, earlier studies report on financial savings, reduced physical restraints, and improved vital signs related to telehealth in nursing homes [11, 12].

There have also been investigations of telemedicine in nursing homes, but few details are known about its effects on nursing care and daily routines of health professionals in the extended care setting [13]. The present study was conducted as a part of the MUT project (Model for the implementation of telemedical care of nursing home residents by outpatient physicians in rural areas) of the German Federal Ministry of Health (grant number: ZMVI1-2520TEL002) [14], which enables telemedical support of health care and supports the necessary technical, legal, training, and organizational conditions. The MUT-project emerged from another project (CAREcomm) [15]. The aim of the project was to identify innovative solutions for the organisation of medical and health care in regions with sparse population, over-ageing and weak infrastructure. The MUT project was performed in the German federal state of Brandenburg, a state of 2.5 million inhabitants with a low physician density. Due to the relatively sparse population, provision of medical care is challenging. The federal state of Brandenburg is characterised by the lowest density of contract physicians in Germany. This is combined with a high utilization rate of outpatient medical care - due, among other things, to the demographic and morbid structure of the population, causing high workloads among health care practitioners and challenges in health care delivery [16]. The MUT project focusses on finding simple and economical telehealth solutions that can be integrated into everyday care with minimal disruption of existing structures, and enabling translation to other institutions potentially benefiting from the MUT experience.

In particular, this study addresses the problems in current home nursing care in rural Germany and how such problems can be alleviated through integrating telehealth into existing structures. We intend the findings of this study to transfer readily to other actors and medical institutions. Thus, we now report on our exploratory qualitative pre-post study based on the following research questions:

- How is medical care currently provided to nursing home residents and where do problems in its delivery occur?
- How can video consultations be used to reduce problems encountered in everyday nursing home care?
- How does present implementation of video consultations impact day-to-day nursing home care delivery?

## Methods

### Study design

To document participants’ perspectives on the implementation of video consultations and to promote their successful implementation, we conducted an exploratory qualitative pre-post study among all parties in the health care process (physicians, nurses and medical technical assistants). We analysed responses to guided, problem-centered interviews [17] using inductive qualitative content analysis to derive replicable and valid overall conclusions of the state of affairs before and after implementation of video consultation. The results were contrasted in a pre-post analysis.

### Participants

The MUT project team organised local information events aimed at physicians and nursing home staff involved in the MUT project [14]. Through the support of volunteers networking in the communities and in the health sector, a total of five information events could be organised in different institutions (e.g. in nursing homes or in town halls). Advertisement yielded recruitment of four physicians, their medical technical assistants and representatives of two nursing homes for the MUT project. In both institutions, all employees were informed about the study and their consent to participate was invited. A total of four physicians, three medical technical assistants and six nurses were recruited for the present study. Detailed information about the sample can be found in section results in Tables 1 and 2.

**Table 1:** Interview partner characteristics prior to implementation of video consultations

Participant ID	Occupation	Sex
MUT_101	Nurse	Female
MUT_102	Medical technical assistant	Female
MUT_103	Nurse	Male
MUT_104	Physician	Female
MUT_105	Medical technical assistant	Female
MUT_106	Physician	Male
MUT_107	Nurse	Female
MUT_108	Physician	Male
MUT_109	Medical technical assistant	Female
MUT_110	Physician	Female
MUT_111	Nurse	Female
MUT_112	Nurse	Female
MUT_113	Nurse	Female

**Table 2:** Interview partner demographics in post-implementation interviews

ID	Occupation	Sex
MUT_201	Physician	Male
MUT_202	Physician	Female
MUT_203	Nurse	Female
MUT_204	Physician	Male
MUT_205	Medical technical assistant	Female
MUT_206	Medical technical assistant	Female
MUT_207	Nurse	Female
MUT_208	Nurse	Female

### Data collection

The preliminary interview guide was developed through participation in two workshops organised by the MUT project. The workshops as part of the MUT project activity aimed at coordinating the project content, objectives, and defining infrastructural requirements for the implementation of video consultations (please refer to supplementary material infrastructural requirements) with the participants of the MUT project. Physicians, nurses, and medical technical assistants talked about problems they encountered in daily health care provision and the processes and structures that might potentially be supported by video consultations. The researchers (SM, FM, GF) were observers at the workshops. Based on their transcripts and memos, they drafted a preliminary interview guide, which was first tested in three pre-interviews with two nurses and one physician, and then adjusted to accommodate their responses. The final interview guide consisted of the following main topics: current medical care provided to nursing home residents, typical problems in everyday care, the potential use of video consultations to reduce these problems, and the impact of implementing video consultations on day-to-day nursing home care delivery (please refer to supplementary material interview guide 1). The second interview guide (post-implementation) addressed the findings of the first interviews and focused on perceived changes in the process (please refer to supplementary material interview guide 2).

In addition, we collected socio-demographic data about the informants, including gender and profession. In order to reduce the risk of infection during the present COVID-19 pandemic, the interviews were conducted via telephone. The baseline phone interviews prior to the implementation of telehealth took place in September 2020, and the post-implementation interviews in November and December of the same year. The interviews were recorded and transcribed verbatim, and the transcripts were pseudonymized according to data protection guidelines.

### Data analysis

Data collection and analysis were conducted in parallel by two researchers (SM, FM), based on Kuckartz’s structured qualitative content analysis [18] using MAXQDA software (Verbi GmbH). Categories were developed inductively, so as to encompass the relevant material in the transcripts using data-driven development of a category system. Next, the category system was applied to the entire interview material. At this stage, data collection had already been completed. To ensure traceability, application of the category system was validated by a member check, whereby the researchers independently applied the developed category system to the entire material (SM, FM). Data collection and analysis were circular and continued until no substantially new findings emerged and theoretical saturation was reached. This manuscript has been compiled in accordance with the Consolidated Criteria for Reporting Qualitative Research (COREQ) [19]. For the presentation of the results, representative quotes of the discussion transcript were selected, translated into English, and are included in this text.

### Ethical considerations

This study was approved by the Ethics Committee of the Brandenburg Medical School Theodor Fontane (E-01-20,200,717). After receiving a study information pack, potential informants were invited to provide a written informed consent prior to participating in the study.

## Results

### Pre-implementation interviews

A total of 13 (ten female, three male) health care professionals participated in the baseline interviews prior to implementation of video consultations. Six participants were nurses from two nursing homes, four were physicians and three were medical technical assistants (see Table 1). Participants were asked to identify current process structures and problems. Furthermore, they were asked to name problems that might be alleviated with video consultations and to express their expectations regarding the advent of telemedicine. The interviews lasted an average of 32 min.

### Depiction of current practice (pre-implementation)

**Fig. 1:**
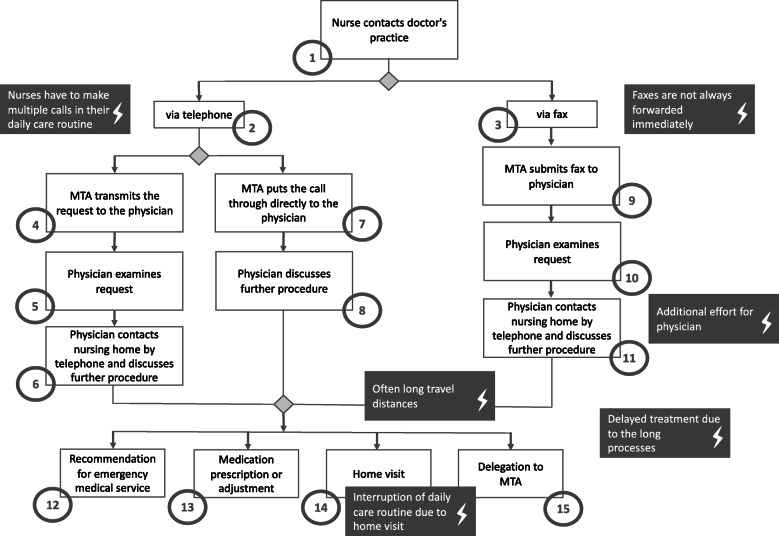
Problems in the current process

To make a request for an acute care visit to the nursing home (1, please refer to Fig. 1 for the flowchart), the nurse contacts the primary care physician’s office via telephone (2) or fax (3). The choice of contact method is at the discretion of the nurse, or following the previously established method of contact between the nursing institution and the medical practice. When contact is made by telephone, the medical assistant at the physician’s surgery takes the call (4) and records the request in writing for transmission to the doctor (5). The physician reviews the request and contacts the nursing facility by phone to discuss the issue (6). In some cases, the medical technical assistant may put the call through directly to the physician (7), such that the physician and the nurse can discuss directly the issue (8). The medical technical assistant decides upon direct or indirect communication based on the current workload in the practice. In summary, the process for planning a face-to-face home visit varied in between participating medical practices. In the case of fax communication from the nursing home, the medical technical assistant receives the fax and transmits it to the physician (9). In this step, the physician checks the request (10) and then contacts the care facility by telephone (11). When subsequently contacting the nursing home, the following scenarios of care are possible: the physician recommends that the ambulance service be contacted for hospital transfer (12), the physician prescribes or adjusts a medication, or (13), schedules a nursing home visit by the physician (14) or by a medical technical assistant (e.g., for blood sampling or wound documentation) (15).

### Problems in the current process

When describing the process for requesting external medical support, the interviewees were asked to name the current obstacles that they perceived in the delivery of care. It emerged that when nurses contact the medical practice by phone, they often had to make several calls to reach the medical technical assistants:

*„Some just answer the phone, some only have certain times when they answer the phone, and then the pressure is rising [...]. So I try to do it on the side, because I can't sit in front [of the phone] the whole time and try again in ten minutes, because then the time for the care runs out again.”* (MUT_101, Pos. 88)

In cases calling for a regular physician home visit, participants frequently noted that nurses would have to interrupt on short notice their daily care routine to be able to participate in the visit.

*„He [the doctor] comes at peak times, when it's lunchtime and you're the only nurse in the living area. I'm either supposed to hand out the medicine or accompany the doctor. I'm a bit grumpy when I have to run away. People need their midday medicine; they're waiting at the lunch table. Then he [the doctor] hangs around for ten minutes, waiting again for you to come.“* (MUT_113, Pos. 20)

When contacted via fax, while faxes arrived directly at the practice, they are not always promptly transmitted to the physician, resulting in delays in arranging an appointment for face-to-face consultation.

*„For example, if the resident has 39.4 fever and somehow it is not possible to see what he/she had. [...]. I called the general practitioner and was told that we should send [the inquiry as] a fax. Then, of course, I get angry. I need an answer NOW and not in the afternoon, when the fever has risen even higher, so I would need someone to give me some information and I can't send a resident to the hospital because of such a temperature. “* (MUT_107, Pos. 6)

In this context, the medical technical assistants have a mediating function. This may not only lead to delays before a consultation can be initiated, but may also result in the loss of information relating to patients and their care.

*„The problem is that a written request always comes in by fax and then we fax it back again […]. That means it is always done by fax, because it is [even more] difficult by phone. This happens during our practice hours, when the MTA [medical technical assistant] can't say much about it, the time is short and that is really a big problem. That's when information gets lost.“* (MUT_106, Pos. 18)

If contact is made by fax, the physician must contact the nursing home again by telephone to arrange an appointment, which requires additional effort on the part of the physician.

*„Yes, for example, everything is currently processed via fax communication. So, when the nursing home makes an inquiry, it is usually made by fax. I usually like to make a phone call, because everything is too vague for me and I want to have it explained. And then I have to answer again by fax. So even though I usually discuss it in advance, I still have to make another written order, for example, so that the colleagues there, the geriatric nurses, have a written order and can work more [assurance] for themselves, and of course that process always duplicates itself somehow.“* (MUT_110, Pos. 8)

In addition, the time expended in visiting the nursing institution, which leads to additional work for the physicians, was also identified as a source of inefficiency. Stakeholders on both sides described that there are often delays in treating nursing home residents because of the often very protracted process to initiate counseling.

*„From the moment when we send out a fax, it actually takes a day before we even get a response.“* (MUT_112, Pos. 26)

The physicians, on the other hand, mentioned that they first have to locate the responsible nursing staff on the unit, which frequently wastes a lot of their time.

*„When I went to the nursing home and had my faxes in hand, [indicating] to whom I had to go, I always went to the ground floor living area and said, “I have this one [patient] and that one [patient], I don't have this and the other one [patient]”. And then I always have to run to the next living area and first of all look for the nursing staff. That is always difficult.“* (MUT_104, Pos. 20)

A detailed illustration of problems reported in the current process can be seen in Fig. 1.

### Sample post-implementation interviews

Eight people (six female, two male) took part in the post-implementation interviews; there were three physicians, three nurses, and two medical assistants due to dropout of one physician, three nurses and one assistant (see in Table 2). The focus of the second session was the analysis of the realisation of video consultations in everyday care, and changes in the process description of the consultations after implementation of telemedicine. Other topics were the problems encountered in implementation, and perceived advantages from telemedicine and its potential for improving the care of patients in nursing homes. The interviews lasted an average of 35 min.

### Depiction of practice after the implementation of videoconferences

Ten weeks after the implementation of videoconferences in everyday care, the stakeholders were asked to describe the revised process: The physician first makes a recommendation for a fixed, weekly time slot for video consultations (1). The video consultations are prescheduled, not arising in response to acute issues. Next, a nurse checks if there are patients who acutely need a physician visit and transmits the patient-relevant information via the video conferencing system 1 day before the scheduled video consultation time slot (2). In this process, all patients are individually assigned an appointment for their respective issues. At the beginning of the video consultation, the nurses and the physician dial into the video consultation software. For this purpose, the nurses use a tablet with the video consultation software installed. The videoconference is carried out from the nurses’ offices, from where the nurses can also view the patient records. First of all, they provide an overview of the patient’s medical report to the physician connected to the system. They initially provide an overview of the patient’s medical record to the physician in order to subsequently discuss the patient’s case. Afterwards, patients come to the nurse’s office if they are mobile. For immobile patients, the video consultation is conducted in the patient’s room.

**Fig. 2:**
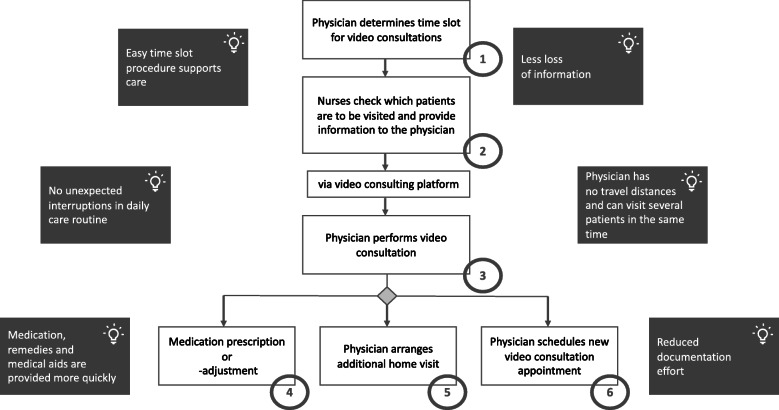
Impact of telemedicine on process post implementation

Following the video consultation (3), the physician can then initiate a medication prescription or adjustment measures (4), arrange an additional face-to-face appointment (5) or another video consultation (6) (see post-implementation flowchart in Fig. 2).

### Impact on current process post implementation

Stakeholders outlined the following changes: A fixed, weekly time slot for video consultations allows scheduled visits to be made for selected patients, as well as enabling ad hoc meetings. This affords the opportunity for nurses to address acute problems without needing to contact the practice again.

*„By creating this one time slot for visits, there is definitely also a certain amount of reliability, planning reliability for the nursing staff. [We are] able to address specific problems in a focused manner, and then together with the patient if necessary.”* (MUT_202, Pos. 35)

The use of video consultations and direct appointment setting eliminates the medical technical assistants’ interface and patient- and care-relevant information is not likely to be lost.

*„It doesn't have to go through an additional person who receives the same information and passes it on to me, and I still then have the nurses describe it to me again in person, because as I said, the loss of information had otherwise already occurred.”* (MUT_202, Pos. 10)

Through the use of electronic visits, continuity of care can be increased and information on patients can be exchanged conveniently. Video consultations reduce interruptions in the day-to-day care of nurses, and physicians no longer have to search for the responsible nurses in the nursing home. If physicians visit the nursing institution spontaneously, this can sometimes lead to longer waiting times for the doctor because the nurses have to accompany them on their ward round at the cost of performing their everyday nursing work. The physicians described that the video consultation hours helped to reduce delays in the daily care routine.

*„I then had first look for the nurse, and had to wait five minutes and then I'm [expletive]. Because I also lacked the time. It's also time pressure. It's an extra task for me that I haven't planned for during the many home visits, and then I have an extra task and I have to wait until one of the ladies or gentlemen [nurses] comes by. Of course, this also affects my mood and puts pressure on my time with the patients. So, I have to say, “Well, come on, guys! Quickly, quickly! I have to move on!”. And now I can do that [visitation] from my armchair with a cup of coffee.“* (MUT_204, Pos. 8)

In addition, travel requirements are eliminated and, if necessary, the attending physicians can care for several patients in the nursing home along with other patients in their regular practice routine during this allotted telehealth time.

*“I just do not have an extra trip and thus also save time. There and back, look at the patient, document the treatment. I have a time saving of almost an hour.”* (MUT_204, Pos. 16)

The nurses transmit the patient-relevant information 1 day before the scheduled appointment to provide the doctors with the necessary information in advance. Through direct communication via video consultation, medical aids and appliances can be prescribed more quickly, and medications can be prescribed or adjusted.

*„So, I say to Dr. W., it's much quicker than if he came here and I discussed everything with him first, and yes, he can decide more quickly who is in immediate need of a visit on site* (MUT_207, Pos. 8)

The documentation in the video consultation software also reduces the organisational and administrative effort, especially for the physicians.

*„Yes, the [need for] faxes is changed, but of course it is still the case that we order the medication in this way; that is normal. But the [need for] communication via faxes about present concerns of the residents, when we needed a consultation, when we needed a new treatment care order or something, that is now all settled in this one appointment. This means that there is really only a single e-mail or invitation at that moment via D. [the video consultation software], [which identifies] what the consultation is about, meaning that - let’s say - seven, eight, nine faxes no longer have to be sent out!“* (MUT_208, Pos. 6)

A detailed illustration of the impact on the process post implementation can be seen in Fig. 2.

## Discussion

This study aimed to explore the capacity of telehealth to support physicians in a rural region, as well as identifying the conditions to ensure successful implementation of this technology into nursing homes in rural Brandenburg. Our findings confirm that telehealth can indeed improve patient care, increase accessibility and continuity of medical care, and reduce the expenditure of effort by nursing staff, technical assistants, and physicians alike. The interviews provide a better understanding of the potential benefits and barriers that should be considered when implementing video consultations in nursing home everyday care.

### Impact on current process

Prior to the implementation of video consultations, the process for scheduling an in-person medical home visit varied between the different medical practices. Our investigation established a process that appears to fit well into everyday practice and has benefits for care delivery. Through previous coordination and scheduling, telehealth time slots were allocated in a structured manner. Our study shows that establishing a convenient time slot procedure is the key factor for a successful and supported expansion of video consultations, which is consistent with the findings in another study [20]. The feeling of not being driven by time constraints led to a more relaxed atmosphere among the doctors and nurses, and simultaneously improved their communication and cooperation. In addition, nurses were no longer interrupted in their work routines, while physicians were spared the extra effort required by travel and seeking out the appropriate nurses on site.

The basic prerequisite for efficient care of nursing home residents is seamless communication between all professionals involved in the care process [21]. In our exploratory qualitative pre-post study, we identified that a slow flow in data exchange, as well as limitations in the accessibility of the individual actors in everyday care, were the main causes of inefficiency in the care process. Due to these factors, clinical processes were slowed down, and additional work was created, which together leads to dissatisfaction in everyday care among the involved healthcare professionals. Our follow-up study indicated a notable improvement in these issues upon telehealth implementation.

Berland and Bentsen stated that medical errors are often caused by a lack of information or poor communication, and only very rarely due to deficient competencies or motivation of the treating and caring professions [22]. Consequently, by improving the exchange of information (as identified in the present study), there can be faster and more efficient provision of care, which is likely to avoid treatment errors, thus leading to greater patient safety. This is consistent with previous findings in the literature [23]. Our study also showed that the elimination of medical technical assistants as the de facto interface between nurses and physicians in the communication process resulted in less loss of clinical information. Thus, the introduction of direct communication through scheduled video consultation leads to faster care and an increase in patient safety.

In addition, participants reported that medications, remedies, and treatment aids were prescribed or adjusted more quickly after telehealth implementation. The communication via video consultation improved the transfer of information and often the speed and accuracy by which health care recommendations could be communicated and implemented. This results from physicians having had the necessary information on specific medical issues beforehand, which could then be addressed directly during the consultation. In addition, informants noted the possibility of holding ad hoc meetings, which, due to time constraints, could rarely be realised during a regular face-to-face visit. This led to improved processing of patient-related needs, whereby patient concerns could be discussed promptly and thus processed more quickly.

The implementation of video consultations has resulted in time savings at various levels; there was a reduction of documentation efforts, savings in travel time for physicians, and facilitation of timely care of patients. By eliminating unnecessary travel, physicians were able to conduct video consultations during the time they might otherwise have been traveling to or from the nursing home, thus increasing their capacity for providing care. These results are line with previous studies [11, 24–26]. Of course, it must be noted that there was initially an additional effort (“learning curve”) required for implementation. However, the benefit to care out-weighted the encumbrances from investing in a tablet or webcam and creating access through a video consultation provider. This was likewise shown in a pilot project during COVID-19 pandemic, where videoconferencing had to be implemented within 24 h to reduce the risk of infection [27]. The circumstances of the COVID-19 pandemic also highlight the importance of providing care via telehealth to non-infected people, which should surely reduce contamination and ensure ongoing medical care in this and future pandemics. Our study also revealed various advantages of video consultations compared to telephone consultations. Health care practitioners are able to visually examine their patients and thus i.e. assess wounds, which is particularly important in nursing home care. Plus, the visual contact creates a more personal and trusting atmosphere and opportunities for reassurance, also with regard to non-verbal communication signs, especially for people with limited verbal communication ability. Furthermore, in Germany, video conferencing offers doctors better reimbursement options than telephone conferencing; allowing the reimbursement of diagnostics and therapy with video conferencing, whereas with telephone consultations, only advice on a disease might be compensated. While the cost of implementing videoconferencing is higher than for teleconferencing, it is still moderate as digitisation in health care progresses. In this case, the expenses include the purchase of a tablet for the nursing home or corresponding hardware in the practices and ensuring adequate internet connection. In the scenario described here, however, the telephone as a medium of communication has not been abolished and continues to serve as a means of contact for urgent cases or similar events.

To obtain a broader integration of telehealth, in this case video consultations, into routine care it is important to emphasize its benefits for the work environment of health care professionals. The present results indicate that telehealth can improve working conditions and lead to higher satisfaction at work, which is consistent with findings of prior studies [28, 29]. To foster the further implementation and adaption of video consultations specifically in nursing home care, we must raise awareness of its benefits for all stakeholders. A broader familiarisation with video consultations would support decisions by institutions to deploy telemedicine services in remote regions [30].

### Strengths and limitations

The qualitative study design with a pre-post data collection has the advantage that the descriptions of the perceived problems with usual processes and the beneficial effects of telehealth can be described in detail, with direct recording of the experiences of the participants through a structured interview approach. The qualitative study design allowed for an in-depth understanding of the impact of implementing video consultations on primary healthcare in nursing homes in rural Germany. Due to the open and exploratory approach, interview partners were able to give considered narrative accounts of their experience.

The study provided information about the impact of video consultations in one rural region in Germany, but further research is needed to examine its relevance in other regions in Germany and other national health systems. At this point, the challenges of video consultation in nursing home care have not been analysed in detail, though we believe this is important and will be considered in our subsequent research efforts. Furthermore, we have not yet investigated its utilization in specialised medical care, such as dental or neurological care. Our recruitment strategy of participants from nursing homes and associated physician practices may have led to a selection bias for those interested in telehealth, and the dropout to follow-up may have lost respondents with more negative experiences. We note that the physician practices and nursing homes experienced staff shortages due to the COVID 19 pandemic, which likely had an impact on the recruitment of participants and facilities, and reduced the sample size especially of nurses for follow-up interviews. We have not offered a cross check of the interview transcripts to the participants.

## Conclusion

To accommodate better the present shortage of physicians and nursing staff, telehealth interventions such as scheduled video consultations, will probably come to complement traditional in-person consultations in future health care delivery. While telehealth cannot entirely replace face-to-face visits, it does offer an alternative mode of service delivery that is amenable to integration into existing structures. Our results suggest that using video consultations in long term nursing care can reduce the burden and expense of travel for physicians and spare nursing staff unnecessary distraction for routine duties, this increasing health care efficiency and productivity.

Further research should focus on patients’ perspectives and explore how older and often multimorbid patient clientele perceive video consultations. In addition, we consider analysis on the applicability of video consultations among non-physician health professionals, e.g., therapists or pharmacists, to be of particular importance to support nursing home care. And finally, there is also a lack of health economic considerations of the use of telemedicine in the nursing home care.

## Supplementary Information

**Additional file 1.** Pre-Implementation Interview Guide.

**Additional file 2.** Post-Implementation Interview Guide.

**Additional file 3.** Supplementary Material: Depiction of infrastructural requirements for the implementation of video consultation identified in the workshops.

## Data Availability

All data relevant to the study are included in the article or uploaded as supplementary information. For further questions regarding the reuse of data, please contact the corresponding author (susann.may@mhb-fontane.de).

## Abbreviations

MUT: Model for the implementation of telemedical care of nursing home residents by outpatient physicians in rural areas
MTA: Medical technical assistant
