# Supplementary material for: Challenges in current nursing home care in rural Germany and how they can be reduced by telehealth - an exploratory qualitative pre-post study
Source: BMC Health Serv Res. 2021 Sep 6;21:925. doi: 10.1186/s12913-021-06950-y (PMC8420146; doi:10.1186/s12913-021-06950-y)
Supplement: Supplementary file 1 — Additional file 1. Pre-Implementation Interview Guide. [file 12913_2021_6950_MOESM1_ESM.docx]

Pre-Implementation Interview Guide

| Guiding Questions | Checkaspects |
| --- | --- |
| -What are the reasons for you to participate in the project MUT?  -What are your expectations when implementing video consultations?  -What do you think will change in your daily practice as a result of participating in the project?  -What changes will have a positive impact on your everyday practice?  -Do you have any concerns? Is there anything that worries you? | Warm-up  identification of expectations, changes and concerns in general |
| -Please describe the current procedure of a doctor’s visit.  -Which typical problems occur in everyday care?  -Which processes or structures can be supported with video consultations?  -What will change in your everyday work?  -How can video consultation make your everyday work easier? What are your expectations? | Potentials of video consultations in medical care routines |
| -What is the current process to get in touch with the doctor's office?  -How do you currently rate the effort involved in communicating with doctors and practice staff?  -What changes in communication do you expect when video consultations are implemented? What will change in detail? | communication |
| -How do you assess the current documentation effort?  -Where could digital interventions reduce the documentation effort?  -Which processes can be supported digitally? | documentation |
| -What advantages do you perceive in the use of video consultation?  -What disadvantages do you perceive?  -What dangers or risks do you perceive in using telemedicine procedures?  -What fears do you have? What could go wrong with the use of telehealth interventions? | Identification of advantages, disadvantages and considerations |
