# Supplementary material for: Challenges in current nursing home care in rural Germany and how they can be reduced by telehealth - an exploratory qualitative pre-post study
Source: BMC Health Serv Res. 2021 Sep 6;21:925. doi: 10.1186/s12913-021-06950-y (PMC8420146; doi:10.1186/s12913-021-06950-y)
Supplement: Supplementary file 2 — Additional file 2. Post-Implementation Interview Guide. [file 12913_2021_6950_MOESM2_ESM.docx]

Post-Implementation Interview Guide

| Guiding Questions | Checkaspects |
| --- | --- |
| -You have participated in a few video consultations by now. Please tell me more about. How did the video consultations proceed? | describing the process of home visits after implementing video consultations |
| -How do you prepare for a video consultation?  -Do you have special times when you implement video consultations (maybe specific time slots for video consultations)?  -How did the scheduling of the visit go? Were there specific time slots that you coordinated together in advance or did rounds take place spontaneously?  -Who initiated visits?  -Did any problems arise? If so, which ones?  -How do the video consultation appointments fit into day to day routines? What do you think is needed to develop routines for video consultation appointments? | preparation/ video consultation appointments |
| -Has the implementation of video consultations changed anything in the communication with the patients? How do you perceive these changes?  -Has the implementation of video consultations changed anything in the communication with other actors? How do you perceive these changes? | communication |
| -How do you end the video consultation?  -How do you document it?  -Did the implementation of video consultations change your documentation? In what form? How did you restructure? | documentation |
| -What has changed in contrast to the usual home visits?  -What differences from the usual home visit have you noticed?  -What advantages did you notice compared to a usual home visit?  -What went better?  -Did you identify any difficulties? If so, where?  -Were there any situations where you would have preferred a standard home visit? Please tell me about it. | changes: usual home visit and video consultation |
