# Supplementary material for: Challenges in current nursing home care in rural Germany and how they can be reduced by telehealth - an exploratory qualitative pre-post study
Source: BMC Health Serv Res. 2021 Sep 6;21:925. doi: 10.1186/s12913-021-06950-y (PMC8420146; doi:10.1186/s12913-021-06950-y)
Supplement: Supplementary file 3 — Additional file 3. Supplementary Material: Depiction of infrastructural requirements for the implementation of video consultation identified in the workshops. [file 12913_2021_6950_MOESM3_ESM.docx]

| **Infrastructural requirements for the implementation of video consultation as described in the workshops**   - Adequate internet connection as key requirement for telehealth usage   - Pre-implementation assessment of infrastructure   - Number of mobile devices in the facility   - Number of simultaneous users in the facility   - Internet coverage of all rooms (if necessary, also paths / corridors)   - Software and hardware status check: State of the art and must support web applications: e.g. computer, laptop or tablet, availability of microphones, loudspeakers and cameras - Administrative confirmation: The video consultation provider has to be certified and approved by the National Association of Statutory Health Insurance Physicians - Further recommendations on video consultation software:   - Mobile device compatibility with intuitive and guided applications   - Integrated appointment function (e.g. physician`s practice & care facility)   - Function for conferencing with up to 5 participants (e.g. teleconsultation, physician`s practice-nursing facility-family members)   - Screen sharing function (e.g. to discuss medical evaluations interactively and visually)   - Screen recording function (e.g. for wound documentation)   - Camera change function (change between front/back or internal/external camera) - Recommendations for initial implementation:   - Identification of key users in the facilities (train the trainer approach)   - Learning by Doing e.g. test phases with experience reports   - Training in the work environment, e.g. test and demo video sessions   - Printable information material e.g. user manuals   - Video tutorials on functions of telehealth applications |
| --- |

*Supplementary Material: Depiction of infrastructural requirements for the implementation of video consultation identified in the workshops*
